# Beyond the Rash: An Atypical Case of Vancomycin-Related Delayed Cutaneous Hypersensitivity

**DOI:** 10.1093/geroni/igaf122.3385

**Published:** 2025-12-31

**Authors:** Paul Chung, Audrey Shawley, Ganeev Singh, Eric Dahms

**Affiliations:** University of California, San Diego School of Medicine, San Diego, California, United States; Scripps Health, San Diego, California, United States; Scripps Mercy Hospital, San Diego, California, United States; Scripps Mercy Hospital, San Diego, California, United States

## Abstract

Severe Cutaneous Adverse Reactions (SCARs), including Drug Reaction with Eosinophilia and Systemic Symptoms (DRESS) and Stevens-Johnson Syndrome (SJS)/Toxic Epidermal Necrolysis (TEN), are life-threatening and often linked to commonly used antibiotics. Older adults are particularly vulnerable due to polypharmacy and age-related pharmacokinetic changes. In this case, a 72-year-old male with basal cell carcinoma and global aphasia from a left parietal stroke developed delayed-onset exfoliative erythroderma. He was hospitalized 5 weeks prior for left leg osteomyelitis, undergoing multiple surgical debridements. Discharged to a skilled nursing facility (SNF) on IV vancomycin and piperacillin-tazobactam, he was switched to daptomycin after a week due to an IV antibiotic shortage. Three weeks later, the SNF noted a worsening full-body, erythematous, non-blanching rash. Despite discontinuing daptomycin for doxycycline, his condition progressed. On admission, he had diffuse petechial and maculopapular lesions with scaling involving his entire body, including the face, palms, and soles, but sparing mucosal membranes. Labs showed mild leukocytosis, elevated neutrophils, no eosinophilia, and elevated CRP/ESR. His rash resolved with antibiotic discontinuation, topical corticosteroids, and IV methylprednisolone. This case underscores the diagnostic challenge of SCARs in polypharmacy-prone older adults, particularly in patients who also face communication barriers secondary to their past medical histories. The delayed onset suggested vancomycin hypersensitivity despite lacking classical DRESS features. SJS/TEN were ruled out due to the timeline. Ultimately, Vancomycin was the most likely culprit for this unusual rash. Clinicians must recognize atypical drug reactions in geriatric patients, where polypharmacy and relevant past medical history complicates diagnosis and management.

